# Identification of meal patterns based on energy intake distribution across the day and their associations with diet quality and body mass index

**DOI:** 10.1265/ehpm.25-00173

**Published:** 2025-10-03

**Authors:** Minami Sugimoto, Keiko Asakura, Sachie Mori, Nana Shinozaki, Kentaro Murakami, Haruhiko Imamura, Yuji Nishiwaki

**Affiliations:** 1 Department of Environmental and Occupational Health, School of Medicine, Toho University, Tokyo, Japan; 2 Institute for Future Initiatives, University of Tokyo, Tokyo, Japan; 3 Department of Preventive Medicine, School of Medicine, Toho University, Tokyo, Japan; 4 Department of Social and Preventive Epidemiology, School of Public Health, The University of Tokyo, Tokyo, Japan; 5 Graduate School of Health and Nutrition Sciences, The University of Nagano, Nagano, Japan

**Keywords:** Energy intake distribution, Body mass index, Food consumption, Nutrient intake, Diet quality, Employees

## Abstract

**Background:**

This cross-sectional study examined meal patterns based on daily energy intake distribution and their associations with nutrient and food intake, diet quality, and body mass index (BMI).

**Methods:**

Body height, weight, habitual dietary intake and the Healthy Eating Index (HEI)-2020 score by eating occasion were assessed using the validated Meal-based Diet History Questionnaire among employees (465 males and 193 females aged 20–75 years) in the Tokyo Metropolitan Area. Meal patterns were extracted based on % energy intake from breakfast, lunch, dinner, and snacks using K-means clustering by sex. Dietary intake, HEI-2020 score, and BMI were then compared between sex-specific meal patterns.

**Results:**

The identified patterns were “large lunch and dinner” (n = 299), “three meals-balanced” (n = 97), and “large dinner” (n = 69) patterns in males and “large dinner” (n = 79); “large afternoon snack” (n = 54) and “large lunch” (n = 60) patterns in females. The HEI-2020 scores were the highest for dinner, followed by breakfast, lunch, and snacks in any meal pattern. Males with the “large dinner” pattern had lower intakes of rice, bread, carbohydrates, dietary fibre, and thiamine; higher intake of alcoholic beverages; and higher HEI-2020 scores than those with other patterns. Females with a “large dinner” pattern had a lower intake of bread, confectionery, total and saturated fats, and carbohydrates; higher intake of fish, meat, and alcoholic beverages; higher HEI-2020 scores; and lower BMI. Thus, a meal pattern with higher energy intake distribution at dinner was associated with higher diet quality among males and females and lower BMI among females in Japanese workers.

**Conclusions:**

These findings suggest that improving the quality of the meal with the highest energy contribution could help enhance overall dietary quality and metabolism.

**Supplementary information:**

The online version contains supplementary material available at https://doi.org/10.1265/ehpm.25-00173.

## Background

Chrono-nutrition is an emerging research field that encompasses eating behaviours such as timing, frequency, and regularity and the distribution of dietary intake throughout the day [1]. Recently, an increasing number of studies have focused on energy intake (EI) distribution [2–13] as a more modifiable factor in eating behaviours [13] and a potential contributor to weight regulation [5]. Previous studies on EI distribution throughout the day have revealed that larger EI at breakfast is associated with better weight status [2–4], whereas larger EI in the evening is associated with a higher risk of obesity [6–8, 10, 11].

These studies mainly captured EI distribution by focusing on the %EI from one eating occasion (such as dinner and within 2 h before bedtime) rather than the meal pattern of the entire day [3–9]. For example, the association between %EI from breakfast and the prevalence of metabolic syndrome was examined without considering EI distribution on other eating occasions such as lunch, dinner, and snacks [3]. Only a few studies have examined meal patterns based on the EI distribution on several eating occasions throughout the day [10–13] and investigated their association with weight status [10–12]. Additionally, only a few studies have investigated the food and nutrient intake and diet quality associated with EI distribution [9–13]. Moreover, previous studies were mainly conducted in Western countries [3, 5–12]. Studies in Asian countries, including Japan, are scarce [4, 13].

The Japanese diet has attracted global interest because of its unique characteristics, such as a higher intake of seaweeds, vegetables, fish, and legumes and a relatively low prevalence of obesity [14, 15]. Additionally, the contribution of breakfast to the Japanese daily EI was higher [16, 17] than that in Western countries such as Denmark, France, and Spain [1, 18]. Furthermore, considering a study that reported comparable nutritional quality of breakfast and dinner among youths with type 1 diabetes in the United States [20], the higher diet quality of dinner compared with breakfast and lunch may be a unique characteristic of Japanese diets [17, 19]. Thus, meal patterns based on EI distribution throughout the day and their association with diet quality and body mass index (BMI) might differ from those in Western countries where studies have reported that higher energy intake in the evening is associated with higher BMI or obesity [6, 10, 11]. However, no study has examined the associations between meal patterns based on EI distribution and weight status in the Japanese population. This study aimed to identify meal patterns based on daily EI distribution and investigate their associations with diet quality and BMI among Japanese workers.

## Methods

### Study design and participants

This cross-sectional study utilised data from a survey conducted in September and October 2022 in the south-eastern ward of the Tokyo metropolitan area, which has a population of 0.7 million [21]. We recruited eight business establishments that were selected from 23 companies certified by the ward for their commitment to health and productivity management. These companies were chosen based on their collaboration with the municipal government and their focus on employee health promotion from a managerial perspective [22]. After receiving invitation emails from ward government officers, companies interested in the study chose to participate. All eight business establishments were small or medium-sized enterprises (≤300 employees), with five having fewer than 100 employees. Few participants worked from home (i.e., teleworking), and none were engaged in shift work. We did not utilise exclusion criteria for recruitment. Sample size calculations were not conducted on a statistical basis before recruitment. The participating companies were recruited to reach the target sample size (n = 800), which was determined based on the number of certified companies and their employees and the feasibility of data collection. Self-administered questionnaires were distributed to all eligible employees in each business establishment (n = 892). The aim and procedure of the study were explained to the participants using a document attached to the questionnaires. Participants agreed to participate in the survey by answering and submitting the questionnaires. Among them, 474 males and 197 females (671 employees in total: response rate = 75%) returned completed questionnaires to the survey office at Toho University.

This study was conducted in accordance with the guidelines of the Declaration of Helsinki. The Ethics Committee of Toho University, Faculty of Medicine, approved all procedures involving humans (approval number: A22028, approval date: 22 July, 2022). The results of this study were reported according to STROBE-nut (Strengthening the Reporting of Observational Studies in Epidemiology—Nutritional Epidemiology guidelines) (online supplementary materials).

### Dietary assessment

Dietary intake was assessed using the Meal-based Diet History Questionnaire (MDHQ), which has been previously described [23]. The MDHQ is a self-administered questionnaire designed to assess the dietary intake over the previous month for each eating occasion (breakfast, morning snack, lunch, afternoon snack, dinner, and night snack). The MDHQ has three parts: part 1 includes quantitative questions regarding the frequency of consumption of common food groups for each eating occasion; part 2 contains questions about the relative frequency of consumption of sub–food groups within common food groups; and part 3 includes questions regarding general eating behaviours. The MDHQ does not collect information on portion sizes except for alcoholic beverages. Estimated energy and nutrient intakes were calculated using an ad hoc computer algorithm based on the 2015 version of the Standard Tables of Food Composition of Japan [24] and the 2011–2012 Food Pattern Equivalents Database in the United States [25].

Nutrient and food intake values were energy-adjusted using the density method (i.e., the amount per 1000 kcal of energy of other nutrients and foods) to minimise the influence of dietary misreporting. The proportions of EI from protein, fat, saturated fat, carbohydrates, and alcohol (for dinner) were calculated for each meal. The relative validity of the MDHQ for nutrient and food intakes was investigated in 222 Japanese adults (n = 111 of each sex) against a four-day diet record [26, 27]. For nutrients, the median value (25^th^ and 75^th^ percentiles) of the Pearson correlation coefficients between the online MDHQ and four-day diet record were 0.54 (0.35–0.57) among females and 0.45 (0.25–0.53) among males. For foods, the median values of the Spearman correlation coefficients were 0.54 (0.38–0.62) for breakfast, 0.30 (0.21–0.42) for lunch, 0.28 (0.20–0.49) for dinner, 0.47 (0.35–0.54) for snacks, and 0.47 (0.42–0.59) for the overall diet among females. The corresponding values for males were 0.60 (0.46–0.67) for breakfast, 0.34 (0.26–0.41) for lunch, 0.24 (0.15–0.38) for dinner, 0.39 (0.33–0.45) for snacks, and 0.49 (0.35–0.59) for the overall diet. For %EI from each eating occasion, Pearson correlation coefficients between the online MDHQ and four-day diet record ranged from 0.44 (dinner) to 0.65 (breakfast) among females and from 0.31 (lunch and dinner) to 0.58 (breakfast) among males [30].

The Healthy Eating Index 2020 (HEI-2020) was used to measure diet quality [28]. The HEI-2020 assesses compliance with the 2015–2020 Dietary Guidelines for Americans [29]. The HEI-2020 consists of nine adequacy components and four moderation components. The nine adequacy components included total fruits (maximum score: 5), whole fruits (5), total vegetables (5), greens and beans (5), whole grains (5), dairy products (5), total protein foods (5), seafood and plant proteins (5), and fatty acids as the ratio of the sum of polyunsaturated fatty acid to saturated fatty acid and of monounsaturated fatty acid to saturated fatty acid (10). The four moderation components are refined grains (10), sodium (10), added sugars (10), and saturated fats (10). The total HEI-2020 score for the overall diet and each eating occasion was calculated based on energy-adjusted values of dietary intake (i.e., amount per 1000 kcal) of energy or percentage of energy, except for the fatty acid component [28]. The dietary intake derived from the MDHQ was used to calculate the score. Spearman correlation coefficients between the HEI-2015 total score by online MDHQ and by diet record were 0.53 for breakfast, 0.43 for lunch, 0.40 for dinner, and 0.49 for the overall diet among females and 0.57 in males [30]. The corresponding values for males were 0.68 for breakfast, 0.43 for lunch, 0.37 for dinner, and 0.57 for the overall diet [30].

Participants were defined as skippers of breakfast, lunch, and dinner when their frequency of eating these meals was less than five times per week in the previous month. Meals that consisted solely of water or beverages were excluded from counting the meal frequency.

### Body mass index

Body height and weight were self-reported as a part of the MDHQ. BMI was calculated as self-reported body weight (in kilograms) divided by the square of body height (in meters). Previous studies have shown that BMI calculated using self-reported height and weight was highly correlated with BMI calculated using measured height and weight [31–34].

### Other variables

The questionnaire collected information on sex, date of birth, smoking habits, family size, the person responsible for meal preparation, job type, working hours per day in the previous month, number of working days per week in the previous month, and proportion of sedentary time during work in the previous month. Age was calculated using the date of birth and the date of answering the questionnaire. Smoking habits were answered using a question with the options “never smoked,” “ex-smoking for more than one year,” “currently smoking (≤20 cigarettes),” and “currently smoking (>20 cigarettes).” Family size was answered by numerous values and then categorised into three groups: 1 (i.e., living alone), 2, and 3 or more. The person responsible for meal preparation could be selected from the following options: myself, my family, myself and my family, and other persons. Job types were assessed with eight options based on the significant job classification by the Ministry of Internal Affairs and Communications [35]: manager, expertise/technical, clerical work, sales service, security officer, manual and labour jobs, and others. Working hours per week in the previous month were calculated by multiplying the number of working days per week by the number of working hours per day. The number of working days at the office and home was summed to determine the total number of working days per week. Working hours per week were categorised into five groups based on a previous systematic review [36]: <35, 35 to 40, 41 to 48, 49 to 54, and ≥55 hours per week [36]. The proportion of sedentary time during work was answered using a question with the options “0–25%,” “26–50%,” “51–75%,” and “76–100%.”

The ratio of EI to the estimated energy requirement (EI:EER) was also calculated as a potential confounder [12, 37, 38]. EI tended to be underreported in obese or overweight participants. In addition, EI misreporting is a confounding factor in the association between eating frequency and adiposity measures [37, 38]. In this study, energy underreporting could lead to the misclassification of meal patterns based on daily EI distribution. Thus, to account for potential reporting bias in EI, EI:EER was adjusted instead of EI itself. The estimated energy requirement was calculated by multiplying the basal metabolic rate by the physical activity level. The basal metabolic rate was calculated using the sex-specific equation for the Japanese population based on age, weight, and height [39]. The “moderately active” physical activity level was assumed for all participants because of a lack of objective information on physical activity by objective measures in this study [40].

### Statistical analysis

All statistical analyses were performed using SAS 9.4 statistical software (version 9.4; SAS Institute Inc.) by sex. All reported P-values were two-tailed, and a P-value < 0.05 was considered statistically significant. Data are presented as means and standard deviation for continuous variables and as numbers and percentages for categorical variables.

The percentage of EI (%EI) from each eating occasion was calculated. Subsequently, K-means clustering was used to extract meal patterns based on the %EI distribution of breakfast, morning snack, lunch, afternoon snack, dinner, and evening snack. The analysis was conducted separately for males and females, considering differences in dietary habits between sex [13, 27]. A three-cluster model was chosen considering the interpretability of the identified patterns [41]. In the analysis with more than four clusters, some patterns were less interpretable and had fewer participants (i.e., representing <10% of the total sample). K-means clustering was performed using the PROC FASTCLUS procedure in SAS 9.4.

For variables of participant characteristics, mean differences among the three patterns were tested using analysis of variance (ANOVA) for continuous variables and χ2 for categorical variables.

Mean differences in food and nutrient intakes and HEI-2020 scores among meal patterns were tested using ANOVA and analysis of covariance (ANCOVA) with the PROC GLM procedure followed by the Tukey-Kramer test. Adjusted means and standard errors (SE) were calculated using the LSMEANS statement with adjustments for age, smoking habits, family size, company, job type, working hours, proportion of sedentary time during work, and EI:EER. Next, BMI was compared among meal patterns in three models using ANOVA and ANCOVA with the PROC GLM procedure followed by the Tukey-Kramer test. In Model 1, covariates were not adjusted. In Model 2, age, smoking habits, family size, company, job type, working hours, the proportion of sedentary time during work, protein intake (% of energy), fat intake (% of energy), alcohol intake (% of energy, continuous), and dietary fibre intake (g/1000 kcal) were adjusted. In the Model 3, the EI:EER was further adjusted. The adjusted means and standard errors of BMI were calculated using the LSMEANS statement.

## Results

**Fig. 1:**
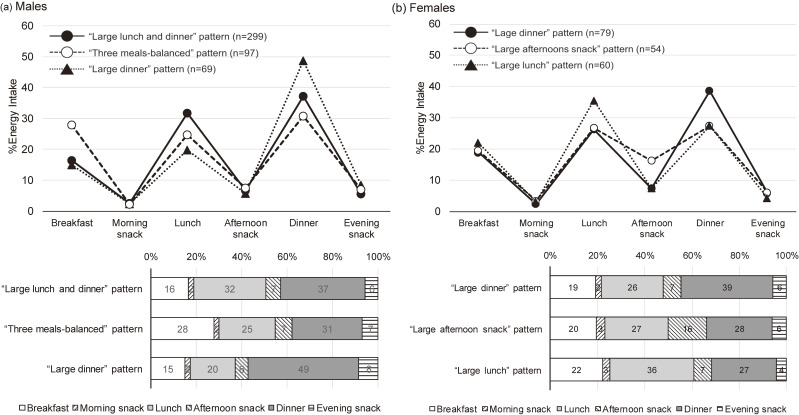
Average of %energy intake from each meal occasion across patterns of %energy intake distribution

One participant was excluded for being outside the target age range (i.e., aged <20 y). Furthermore, 12 participants were excluded due to missing data on either smoking habits, working hours, or both. Thus, 658 workers (465 males and 193 females) were included in the analysis. Using K-means clustering, three meal patterns based on the %EI distribution were derived for males and females. Figure 1 shows the %EI distribution for each pattern. Each pattern was labelled based on the eating occasions with a higher %EI. For males, “large lunch and dinner” pattern (n = 299) with a low %EI from breakfast (16%) and a high %EI from lunch (32%) and dinner (37%); “three meals-balanced” pattern (n = 97) with equal %EI for all three meals (25–31%); and “large dinner” pattern (n = 69), with a low %EI from breakfast (15% on average) and lunch (20%) and a high %EI for dinner (49%). For females, “large dinner” pattern (n = 79), with a higher %EI from dinner (39% on average) than breakfast (19%) and lunch (26%); “large afternoon snack” pattern (n = 54) with equal %EI from the three main meals (20–28% on average) but having higher %EI from afternoon snack (16% on average) than other patterns (7% on average); and “large lunch” pattern (n = 60) with higher %EI from lunch (36% on average) than breakfast (22%) and dinner (27%).

In males, the participants with the “large lunch and dinner” pattern tend to be younger, be sales service workers, skip breakfast, live alone, prepare their own meals, and work longer hours (Table 1). The participants in the “three meals-balanced” pattern tended to be older and clerical workers. The participants in the “large dinner” pattern tended to be older, to be sales service workers, and to skipped breakfast and lunch. There was no difference between patterns in other basic characteristics except for EI:EER and the proportion of dinner skippers in females. Females with the “large lunch” pattern had lower EI:EER than those with other patterns.

**Table 1:** Characteristics of study participants according to meal patterns based on %energy intake distribution

	**Males (n = 465)**							**Females (n = 193)**						

	**“Large lunch and dinner” pattern (n = 299)**		**“Three meals-balanced” pattern ** **(n = 97)**		**“Large dinner” pattern ** **(n = 69)**		P*	**“Large dinner” pattern ** **(n = 79)**		**“Large afternoon snack” pattern (n = 54)**		**“Large lunch” pattern (n = 60)**		P*
Age (y)	38.6	13.8	47.0	14.1	45.8	11.8	<.0001	39.5	12.9	37.6	12.0	34.6	12.9	0.08
Overall energy intake (kcal/d)	1969	600	2027	430	1944	483	0.58	1501	368	1577	353	1414	380	0.06
EI:EER	0.74	0.24	0.76	0.18	0.74	0.20	0.70	0.78	0.21	0.79	0.19	0.70	0.20	0.02
Meal skippers (N, %)†														
Breakfast	150	(50)	5	(5)	44	(64)	<.0001	33	(42)	16	(30)	18	(30)	0.23
Lunch	8	(3)	11	(11)	28	(41)	<.0001	6	(8)	3	(6)	1	(2)	0.29
Dinner	13	(4)	9	(9)	2	(3)	0.11	0	(0)	5	(9)	7	(12)	0.01
Smoking habits (N, %)							0.0003							0.12
Never smoked	115	(38)	39	(40)	17	(25)		49	(62)	39	(72)	49	(82)	
Ex-smoking for ≥1 y	84	(28)	40	(41)	15	(22)		18	(23)	5	(9)	7	(12)	
Currently smoking (≤20 cigarettes)	89	(30)	16	(16)	31	(45)		11	(14)	9	(17)	4	(7)	
Currently smoking (>20 cigarettes)	11	(4)	2	(2)	6	(9)		1	(1)	1	(2)	0	(0)	
Family size‡							0.003							0.22
1 (i.e., living alone)	98	(33)	17	(18)	11	(16)		24	(30)	15	(28)	26	(43)	
2	72	(24)	21	(22)	18	(26)		25	(32)	19	(35)	11	(18)	
3 or more	129	(43)	59	(61)	39	(57)		28	(35)	20	(37)	22	(37)	
Person in charge of meal preparation							0.01							0.62
By myself	112	(37)	23	(24)	15	(22)		56	(71)	32	(59)	38	(63)	
My family	127	(42)	56	(58)	35	(51)		10	(13)	13	(24)	13	(22)	
By myself and my family equal	53	(18)	18	(19)	15	(22)		12	(15)	9	(17)	8	(13)	
Other person	3	(1)	0	(0)	3	(4)		1	(1)	0	(0)	1	(2)	
No response	4	(1)	0	(0)	1	(1)		0	(0)	0	(0)	0	(0)	
Company							0.18							0.77
A	136	(45)	34	(35)	20	(29)		21	(27)	19	(35)	28	(47)	
B	30	(10)	10	(10)	5	(7)		4	(5)	2	(4)	2	(3)	
C	36	(12)	16	(16)	10	(14)		14	(18)	11	(20)	7	(12)	
D	51	(17)	21	(22)	17	(25)		21	(27)	11	(20)	10	(17)	
E	18	(6)	7	(7)	5	(7)		3	(4)	3	(6)	3	(5)	
F	18	(6)	4	(4)	7	(10)		4	(5)	1	(2)	1	(2)	
G	5	(2)	4	(4)	1	(1)		3	(4)	1	(2)	3	(5)	
H	5	(2)	1	(1)	4	(6)		9	(11)	6	(11)	6	(10)	
Job type							0.40							0.19
Manager	68	(23)	23	(24)	15	(22)		4	(5)	1	(2)	4	(7)	
Expertise/Technical	33	(11)	14	(14)	13	(19)		5	(6)	6	(11)	5	(8)	
Clerical work	50	(17)	22	(23)	10	(14)		38	(48)	32	(59)	35	(58)	
Sales service	109	(36)	27	(28)	24	(35)		15	(19)	10	(19)	14	(23)	
Security officer	2	(1)	0	(0)	1	(1)		2	(3)	1	(2)	0	(0)	
Manual and labor jobs	36	(12)	9	(9)	6	(9)		13	(16)	2	(4)	2	(3)	
Other	1	(0)	2	(2)	0	(0)		2	(3)	2	(4)	0	(0)	
Working hour (h/week)							0.49							0.18
<35	3	(1)	1	(1)	2	(3)		4	(5)	7	(13)	2	(3)	
35–40	102	(34)	39	(40)	27	(39)		38	(48)	25	(46)	25	(42)	
41–48	63	(21)	20	(21)	14	(20)		22	(28)	10	(19)	12	(20)	
49–54	75	(25)	28	(29)	15	(22)		11	(14)	6	(11)	13	(22)	
≥55	56	(19)	9	(9)	11	(16)		4	(5)	6	(11)	8	(13)	
Proportion of sedentary time during work§							0.48							0.26
0–25%	37	(12)	9	(9)	3	(4)		6	(8)	3	(6)	2	(3)	
26–50%	73	(24)	28	(29)	20	(29)		12	(15)	4	(7)	9	(15)	
51–75%	107	(36)	30	(31)	29	(42)		25	(32)	12	(22)	23	(38)	
76–100%	78	(26)	26	(27)	16	(23)		36	(46)	33	(61)	25	(42)	
Body height (cm; mean, SD)	172.5	5.8	172.2	6.1	171.9	5.1	0.74	157.9	5.6	157.9	5.2	158.5	5.0	0.77
Body weight (kg; mean, SD)	69.5	11.2	72.1	12.5	69.5	12.3	0.15	50.7	5.7	53.9	7.0	54.5	8.0	0.002
Weight status (N, [%])							0.43							0.02
Underweight	13	(4)	2	(2)	3	(4)		14	(18)	8	(15)	5	(8)	
Normal	206	(69)	60	(62)	47	(68)		64	(81)	38	(70)	47	(78)	
Overweight	80	(27)	35	(36)	19	(28)		1	(1)	8	(15)	8	(13)	

EI:EER, ratio of energy intake to estimated energy requirements.

*P values by analysis of variance for continuous variables and χ2 for categorical variables. P < 0.05 was considered to indicate statistical significance.

† Meal skippers are those who ate breakfast [lunch, or dinner], including some solid foods less than five times per week in the previous month.

‡ One man and three women did not answer the question.

§ Nine men and three women did not answer the question.

In comparison with food and nutrient intakes, males with the “large lunch and dinner” pattern had higher intakes of rice, noodles, meat, carbohydrates, thiamine, zinc, and copper and lower intakes of confectionaries and alcoholic beverages, and calcium than other patterns (Table 2). Those with the “three-meals balanced” pattern had higher intakes of rice, bread, eggs, dairy products, carbohydrates, dietary fibre, calcium, iron, zinc, and copper and lower intakes of noodles, meat, confectionaries, alcoholic beverages and calcium. Those with the “large dinner” pattern had higher intakes of alcoholic beverages and lower intakes of rice, bread, carbohydrates, dietary fibre, and thiamine. Females with the “large dinner” patterns had higher intakes of fish, meat, alcoholic beverages, protein, niacin, and vitamin B_6_ and lower intakes of bread, confectionaries, total fat, saturated fat, and carbohydrates. Those with the “large afternoon snack” pattern had higher intakes of confectionaries, total fat, and saturated fat, and lower intakes of potatoes, fish, meat, alcoholic beverages, protein, niacin, and vitamin B_6_. Those with the “large lunch” pattern had a higher intake of bread, potatoes, protein, and carbohydrates and lower intakes of confectionaries, alcoholic beverages, and saturated fat. The “large lunch” pattern tended to have a higher rice and sweetened beverages intakes than other patterns, but there was no statistical significance.

**Table 2:** Comparison of food and nutrient intake between meal patterns based on %energy intake distribution

	**Males (n = 465)**							**Females (n = 193)**						

	**“Large lunch and dinner” pattern (n = 299)**		**“Three meals-balanced” pattern ** **(n = 97)**		**“Large dinner” pattern (n = 69)**		P*	**“Large dinner” pattern (n = 79)**		**“Large afternoon snack” pattern (n = 54)**		**“Large lunch” ** **pattern ** **(n = 60)**		P*
Food intake (g/1000 kcal)														
Rice	144.8a	3.05	140.0a	5.45	113.4b	6.38	<.0001	116.1	5.5	99.0	6.6	119.8	6.5	0.08
Bread	22.9a,b	0.94	27.2a	1.68	19.9b	1.97	0.02	20.1a	1.9	22.0a,b	2.2	28.0b	2.2	0.02
Noodle	51.8a	1.62	42.6b	2.90	48.2a,b	3.39	0.02	47.2	3.2	41.2	3.8	50.9	3.8	0.31
Potato	12.5	0.43	12.1	0.77	13.0	0.90	0.74	14.4ab	0.9	12.7a	1.1	16.8b	1.1	0.03
Legume	16.5	0.66	18.2	1.17	18.0	1.37	0.38	21.5	1.5	20.1	1.8	20.8	1.8	0.85
Total vegetables	64.8	2.20	66.7	3.94	70.9	4.61	0.49	93.1	5.1	82.8	6.0	85.2	5.9	0.31
Fruits	30.8	0.94	33.6	1.67	31.5	1.96	0.35	38.8	2.6	39.9	3.1	40.2	3.1	0.95
Fish	20.2	0.63	20.0	1.13	21.2	1.32	0.75	22.5a	1.3	15.6b	1.5	20.6a,b	1.5	0.01
Meat	34.7a	0.74	30.9b	1.32	32.3a,b	1.55	0.03	34.3a	1.4	29.2b	1.7	29.5a,b	1.6	0.04
Eggs	12.5a	0.40	15.2b	0.72	10.9a	0.84	0.0002	13.1	0.8	12.5	1.0	12.7	0.9	0.74
Dairy products	41.3a,b	1.75	58.3a	3.13	44.9b	3.67	<.0001	61.6	4.4	66.7	5.3	65.6	5.2	0.85
Confectionaries	28.5a	0.93	33.4b	1.66	28.6a,b	1.94	0.03	31.0a	2.1	56.3b	2.5	37.3a	2.5	<.0001
Fruits juice	82.1	3.02	85.5	5.39	77.3	6.32	0.62	58.0	3.9	54.7	4.7	66.6	4.6	0.23
Alcoholic beverages	107.3a	7.8	83.5a	13.8	206.8b	16.2	<.0001	143.4a	14.3	65.9b	17.0	44.3b	16.8	<.0001
Sweetened beverages	77.0	3.43	61.7	6.12	72.1	7.17	0.10	52.7	5.3	65.5	6.3	71.4	6.2	0.06
Tea and coffee	500	13.0	470	23.2	504	27.2	0.51	600.5	50.0	702.0	59.6	712.8	58.7	0.23
Nutrient intake														
Protein (%energy)	12.1	0.10	12.3	0.18	11.7	0.21	0.08	12.7a	0.2	12.0b	0.2	12.9a	0.2	0.01
Total fat (%energy)	23.7	0.26	24.2	0.47	23.1	0.55	0.28	25.8a	0.5	28.5b	0.6	26.5a,b	0.6	0.01
Saturated fatty acid (%energy)	7.4	0.10	7.7	0.18	7.3	0.21	0.28	8.09a	0.2	10.1b	0.3	8.41a	0.3	<.0001
Carbohydrate (%energy)	56.4a	0.41	56.2a	0.74	51.0b	0.86	<.0001	52.3a	0.7	54.8a	0.8	57.3b	0.8	<.0001
Dietary fibre (g/1000 kcal)	5.90a,b	0.07	6.22a	0.13	5.65b	0.15	0.01	6.75	0.15	6.72	0.18	7.09	0.17	0.17
Vitamin A (µg/1000 kcal)	177.0	3.93	186.4	7.02	178.5	8.22	0.52	215.8	12.8	217.4	15.2	229.9	15.0	0.56
Thiamine (mg/1000 kcal)	0.44a	0.005	0.42a,b	0.009	0.40b	0.010	0.003	0.46	0.01	0.44	0.01	0.46	0.01	0.36
Riboflavin (mg/1000 kcal)	0.6	0.006	0.6	0.011	0.6	0.013	0.23	0.65	0.02	0.70	0.02	0.69	0.02	0.11
Niacin (mg/1000 kcal)	8.5	0.10	8.1	0.18	8.7	0.21	0.07	9.47a	0.2	8.30b	0.3	8.72a,b	0.3	0.002
Vitamin B_6_ (mg/1000 kcal)	0.55	0.007	0.54	0.012	0.56	0.014	0.37	0.62a	0.0	0.55b	0.0	0.58a,b	0.0	0.003
Vitamin B_12_ (µg/1000 kcal)	2.2	0.06	2.2	0.11	2.3	0.13	0.64	2.39	0.13	1.95	0.15	2.24	0.15	0.12
Folate (µg/1000 kcal)	131.1	1.94	136.0	3.47	131.9	4.06	0.48	153.8	5.0	149.4	5.9	159.1	5.8	0.44
Vitamin C (mg/1000 kcal)	43.9	0.87	45.2	1.55	43.4	1.82	0.69	47.9	1.9	46.1	2.2	50.0	2.2	0.38
Sodium (mg/1000 kcal)	2049	23.9	1987	42.7	1921	50.1	0.06	2162	59	2012	71	2229	69.6	0.10
Potassium (mg/1000 kcal)	1193	13.0	1229	23.1	1199	27.1	0.40	1351	28	1305	33	1352	33.0	0.27
Calcium (mg/1000 kcal)	223a	3.24	245b	5.78	230a,b	6.77	0.004	267	7.2	291	8.6	281	8.4	0.16
Magnesium (mg/1000 kcal)	132.2	1.43	135.5	2.55	134.1	2.98	0.51	149	3.0	144	3.5	147	3.5	0.37
Iron (mg/1000 kcal)	3.28a,b	0.03	3.39a	0.06	3.14b	0.07	0.03	3.60	0.07	3.68	0.09	3.75	0.09	0.33
Zinc (mg/1000 kcal)	3.8a	0.03	3.8a	0.06	3.6b	0.07	0.006	3.87	0.06	3.73	0.07	3.97	0.07	0.04
Copper (mg/1000 kcal)	0.55a	0.005	0.56a	0.008	0.51b	0.01	0.0003	0.57	0.009	0.60	0.011	0.60	0.011	0.03

Values are mean estimates and SE adjusted for age (year, continuous), smoking habits (never smoked, ex-smoking for ≥1 y, currently smoking [≤20 cigarettes], currently smoking [>20 cigarettes], family size (missing, 1, 2, ≥3), job type (manager, expertise/technical, clerical work, sales service, security officer, manual and labour jobs, other), company, working hours (<35, 35–40, 41–48, 49–54, ≥55), and proportion of sedentary time during work (missing, 0–25%, 26–50%, 51–75%, 76–100%), and the ratio of energy intake to expected energy requirement (continuous). Mean values with different letters were significantly different by the post-hoc Tukey test (P < 0.05).

*P values by analysis of variance. P < 0.05 was considered to indicate statistical significance.

Overall, the HEI-2020 score for dinner was the highest, followed by breakfast, lunch, and snacks (Table 3). When comparing the HEI-2020 score of overall diet between patterns, males with “large dinner” patterns (adjusted mean ± SE: 51.0 ± 0.9) had a higher score than the “large lunch and dinner” pattern (47.9 ± 0.5). The HEI-2020 score for dinner in males with the “large dinner” pattern was higher than those with the “large lunch and dinner” pattern. Females with the “large dinner” (52.2 ± 1.0) and “large lunch” (49.1 ± 1.2) patterns had higher HEI-2020 scores for overall diet than those with the “large afternoon snack” pattern (44.8 ± 1.2). Similarly, the HEI-2020 score for snacks in those with the “large dinner” and “large lunch” patterns was higher than those with the “large afternoon snack” patterns.

**Table 3:** Healthy Eating Index-2020 score between meal patterns based on %energy intake distribution

	**Males (n = 465)**							**Females (n = 193)**						

	**“Large lunch and dinner” pattern (n = 299)**		**“Three meals-balanced” pattern (n = 97)**		**“Large dinner” pattern ** **(n = 69)**		P*	**“Large dinner” pattern ** **(n = 79)**		**“Large afternoon snack” pattern (n = 54)**		**“Large lunch” ** **pattern (n = 60)**		P*
Total score (whole day) (max: 100)	47.9a	0.5	49.5a,b	0.8	51.0b	0.9	0.009	52.2a	1.0	44.8b	1.2	49.1a	1.2	<.0001
Sub score (whole day) (maximum score)														
Total fruits (5)	2.34	0.06	2.57	0.10	2.32	0.12	0.11	2.35	0.11	2.43	0.14	2.63	0.13	0.30
Whole fruits (5)	2.50	0.07	2.77	0.12	2.52	0.14	0.14	3.05	0.14	3.08	0.17	3.20	0.16	0.78
Total vegetables (5)	3.77	0.07	3.94	0.12	3.95	0.14	0.33	4.41	0.12	4.09	0.14	4.01	0.14	0.06
Green and beans (5)	1.65a,b	0.08	2.00a	0.14	1.45b	0.17	0.03	2.38	0.18	1.87	0.21	2.07	0.21	0.15
Whole grains (5)	1.77	0.13	1.93	0.24	1.67	0.28	0.76	1.83	0.23	1.88	0.27	2.30	0.27	0.40
Dairy (10)	1.74a	0.07	2.27b	0.13	2.17b	0.15	0.001	2.52	0.16	2.68	0.19	2.64	0.19	0.81
Total protein foods (5)	3.98	0.05	3.94	0.09	3.93	0.11	0.88	4.20a	0.10	3.74b	0.12	3.96a,b	0.12	0.02
Seafood and plant proteins (5)	4.48	0.05	4.55	0.08	4.68	0.10	0.19	4.63	0.09	4.35	0.11	4.56	0.11	0.13
Fatty acid (10)	5.07	0.13	4.86	0.24	5.02	0.28	0.76	5.20a	0.27	2.83b	0.32	4.80a	0.32	<.0001
Refined grains (10)	1.25a	0.13	1.21a	0.23	2.80b	0.27	<.0001	2.63a	0.28	2.33a,b	0.34	1.34b	0.33	0.02
Sodium (10)	1.53a	0.14	1.87a,b	0.25	2.37b	0.29	0.03	1.26	0.24	1.78	0.29	1.17	0.29	0.26
Added sugar (10)	8.33	0.12	8.27	0.21	8.78	0.24	0.20	8.70a	0.22	6.58b	0.27	7.56c	0.26	<.0001
Saturated fats (10)	9.52	0.07	9.28	0.13	9.37	0.15	0.24	8.99a	0.21	7.14b	0.25	8.87a	0.24	<.0001
Total score of each meal occasion (max: 100)														
Breakfast	46.8	0.5	49.1	0.9	47.5	1.1	0.09	50.7	1.0	49.5	1.2	49.4	1.2	0.68
Lunch	45.0	0.5	46.9	0.8	44.0	1.0	0.050	48.9	1.0	46.1	1.2	47.7	1.1	0.17
Dinner	53.1a	0.4	54.6a,b	0.8	55.9b	0.9	0.02	57.1	0.9	56.0	1.0	54.4	1.0	0.15
Snacks	43.4	0.4	42.2	0.7	44.5	0.8	0.08	40.8a	0.8	32.2b	0.9	40.6a	0.9	<.0001

Values are mean estimates and SE adjusted for age (year, continuous), smoking habits (never smoked, ex-smoking for ≥1 y, currently smoking [≤20 cigarettes], currently smoking [>20 cigarettes]), family size (missing, 1, 2, ≥3), job type (manager, expertise/technical, clerical work, sales service, security officer, manual and labour jobs, other), company, working hours (<35, 35–40, 41–48, 49–54, ≥55), and proportion of sedentary time during work (missing, 0–25%, 26–50%, 51–75%, 76–100%), and the ratio of energy intake to expected energy requirement (continuous). Mean values with different letters were significantly different by the post-hoc Tukey test (P < 0.05).

*P values by analysis of variance. P < 0.05 was considered to indicate statistical significance.

In males, no significant difference was observed in BMI among patterns in both the crude and the adjusted model (Table 4). Adjusted mean and SE of BMI were 23.5 ± 0.20 for the “large lunch and dinner” pattern, 24.1 ± 0.35 for the “three meals-balanced” pattern, and 23.2 ± 0.43 for the “large dinner” pattern in Model 3. In females, the “large afternoon snack” pattern (21.7 ± 0.37) and the “large lunch” (21.6 ± 0.35) had higher BMI than the “large dinner” (20.3 ± 0.31) pattern.

**Table 4:** Comparison of body mass index (kg/m^2^) between meal patterns based on %energy intake distribution

**Model**	**Unit**	**Males (n = 465)**							**Females (n = 193)**						

		**“Large lunch and dinner” pattern (n = 299)**		**“Three meals-balanced” pattern ** **(n = 97)**		**“Large dinner” pattern (n = 69)**		P*	**“Large dinner” pattern (n = 79)**		**“Large afternoon snack” pattern (n = 54)**		**“Large lunch” pattern (n = 60)**		P*
Model 1†	Mean, SD	23.4	0.2	24.3	0.4	23.5	0.4	0.08	20.3a	1.9	21.6b	3.0	21.7b	3.0	0.002
Model 2‡	Adjusted mean, SE	23.5	0.21	24.0	0.37	23.4	0.45	0.49	20.3a	0.31	21.6b	0.38	21.7b	0.36	0.007
Model 3§	Adjusted mean, SE	23.5	0.20	24.1	0.35	23.2	0.43	0.26	20.3a	0.31	21.7b	0.37	21.6b	0.35	0.01

Mean values with different letters were significantly different by the *post-hoc* Tukey test (P < 0.05)

* P values by analysis of variance for model 1 and analysis of covariance for models 2 and 3. P < 0.05 was considered to indicate statistical significance

† Crude model.

‡ Adjusted for age (year, continuous), smoking habits (never smoked, Ex-smoking for ≥1 y, currently smoking [≤20 cigarettes], currently smoking [>20 cigarettes]), family size (missing, 1, 2, ≥3), job type (manager, expertise/technical, clerical work, sales service, security officer, manual and labor jobs, other), working hours (<35, 35–40, 41–48, 49–54, ≥55), and proportion of sedentary time during work (missing, 0–25%, 26–50%, 51–75%, 76–100%), protein intake (% of energy, continuous), fat intake (% of energy, continuous), total carbohydrate intake (% of energy, continuous), alcohol intake (% of energy, continuous), and dietary fibre intake (g/1000 kcal, continuous).

§ Adjusted for variables used in model 2 and the ratio of energy intake to expected energy requirement (continuous)

## Discussion

This study found that participants with meal patterns featuring a higher %EI at dinner showed higher diet quality than those with other patterns in both males and females. Additionally, no difference was observed in the BMI between males with different meal patterns. However, females with the “large dinner” pattern had lower BMI than those with the “large afternoon snack” and “large lunch” patterns. To our knowledge, this study is the first to investigate the association of meal patterns based on %EI distribution with diet quality and BMI among the Japanese population.

Our results were inconsistent with those of previous studies that revealed that a higher distribution of EI earlier in the day was associated with greater weight loss [2, 3] and a lower risk of obesity [4, 10]. Consuming more energy in the evening was associated with a higher risk of obesity [6, 7] and increased BMI [8]. Moreover, a higher %EI at lunch was associated with a lower risk of weight gain [5], and evenly spaced, energy-balanced eating occasions were associated with lower BMI and odds of obesity compared with other patterns [11]. A possible reason for this inconsistency with previous studies might be the differences in dietary habits associated with EI distribution among participants. A German study found that higher evening EI was associated with lower diet quality [10], whereas an Australian study showed that a larger breakfast size was associated with higher diet quality, but not with BMI [9]. However, in this study, meal patterns with a higher %EI at dinner had higher HEI-2020 scores than other patterns. In addition, the HEI-2020 scores for lunch and snacks were lower than those for dinner across all patterns. These results suggest that the diet quality of eating occasions with a higher %EI within the day, particularly the main meal of the day, is important in addition to EI distribution for preventing obesity, especially in females. Improving the diet quality of lunch, which had a higher %EI but the lowest diet quality among the three main meals, could enhance overall diet quality. Many previous studies on EI distribution did not detail food and nutrient intakes or diet quality, making it difficult to discuss meal-specific dietary characteristics. Future studies should describe these dietary characteristics to interpret their results better.

There was no significant difference in BMI between %EI distribution patterns in males in this study. Possible reasons include participant characteristics, energy expenditure, meal-skipping frequency, and small differences in diet quality between patterns. Males with the “large lunch and dinner” patterns were younger, whereas those with “three-meals balanced” patterns were older. According to the national survey, older Japanese individuals (40–59 y) had higher BMI than younger individuals (20–39 y) in males and females [14]. Age was adjusted for in the analysis, but the effect of age differences may not have been fully adjusted. Additionally, information on physical activity was lacking. Differences in physical activity levels may have confounded the association between %EI distribution patterns and BMI. Moreover, participants in the “large lunch and dinner” and “large dinner” patterns often skipped breakfast or lunch, potentially lowering their total energy intake. Additionally, the small differences in HEI-2020 scores between the three patterns (<4 points) could explain the lack of substantial BMI differences. Each pattern had favourable and unfavourable aspects of dietary intake in males. For example, males with the “dinner” pattern had a higher HEI-2020 score but higher alcoholic intake and lower dietary fibre. Those with the “three meals-balanced” pattern had higher micronutrient intakes but also higher refined grain intake. Thus, dietary improvements are needed for males across all patterns.

The higher BMI in females with the ‘large afternoon snack’ pattern compared with the ‘large dinner’ pattern might be attributed to their higher intake of confectionaries and sweetened beverages, though the latter was insignificant. A previous systematic review showed that a higher intake of confectionaries and sweetened beverages was associated with higher BMI [42]. Another study showed that a higher % of snacking energy from desserts and sweets was associated with higher BMI [43]. Regarding the “large lunch” pattern, the higher BMI might be caused by a higher intake of refined cereals and sweetened beverages. Studies have shown positive associations between refined grain intake and weight gain [44, 45] as well as the incidence of overweight/obesity [46]. In addition, a previous systematic review observed a positive association between white rice intake and the risk of all overall chronic diseases in females [47]. The adverse health effects of unhealthy foods, such as confectionaries and carbohydrate-rich foods, may be amplified if consumed on eating occasions with a larger %EI but low diet quality. Altogether, a higher %EI from snacks and lunch might be associated with higher BMI owing to lower diet quality on these eating occasions. Additionally, reverse caution may explain the higher BMI in females with the “large lunch” pattern, as they might eat more at lunch and less at dinner, believing that large energy intake in the evenings contributes to weight gain.

This study has some limitations. First, the study population was not randomly selected nor nationally representative. It consisted of staff members who worked at only eight offices in the Tokyo Metropolitan Area, with 70% of the participants being males. Although distinct patterns of EI distribution were observed among females, their small sample size provided little statistical power. Thus, the generalisability of our results to other Japanese populations may be low, and further studies in other working populations with diverse samples across geographic regions and socioeconomic backgrounds are needed. Secondly, BMI was calculated based on self-reported body weight and height, whose accuracy using the MDHQ is unknown. A previous scoping review of studies in Japan showed that self-reported body weight tends to be under-reported, and self-reported body height tends to be over-reported [48]. Thus, misreporting body weight and height might have weakened the association between %EI distribution and BMI. However, high correlations were observed between BMI based on measured and self-reported values [31–34], making self-reported BMI acceptable for comparing participants’ weight statues. Third, dietary intake was self-reported. It is widely observed that energy intake tends to be underestimated among obese or overweight participants [40]. The MDHQ is a validated questionnaire for assessing nutrient and food intake and diet quality [30, 31, 34]; however, energy intake tends to be under-reported [30]. Nonetheless, we used the %EI from each eating occasion rather than the crude value of reported energy intake in our analysis, minimising the effect of under-reporting of energy intake on our results. Fourth, information on potential confounding factors, such as physical activity level, socio-economic status, medical history, psychological stress, and sleep quality, was not fully collected. For example, if certain %EI distribution patterns are associated with higher physical activity levels, those patterns might have a lower BMI. However, physical activity levels at work were likely similar across patterns, as job types did not differ among those with different patterns. Moreover, some participants might not have had intensively higher occupational and physical activity levels than others, as the usual work of most participants was physically inactive (such as desk work or standing jobs). Not considering medical history might not have biased the results since our participants were relatively healthy working adults, and the number with a medical history was likely small. Further studies are needed to investigate whether these factors confound the association between %EI distributions and BMI. Lastly, due to the observational and cross-sectional study design, it cannot establish causal relationships between meal patterns and body mass index. There could be causal relationships between meal patterns, body mass index and diet quality. Longitudinal or interventional studies would be necessary to confirm any causal associations.

In conclusion, males and females with a higher %EI from dinner had higher HEI-2020 scores, whereas men with a higher %EI from lunch and dinner and females with a higher %EI from snacks had lower HEI-2020 scores. Considering the lower diet quality for lunch and snacks compared with other eating occasions among the participants, a larger proportion of energy intake from lunch and snacks might be associated with a higher BMI, especially in females.

## Abbreviations

BMI: body mass index
EI: energy intake
EI:EER: energy intake to the estimated energy requirement
HEI: Healthy Eating Index
MDHQ: Meal-based Diet History Questionnaire

## Supplementary information

**Additional file 1:** **Table 1.** STROBE-nut: An extension of the STROBE statement for nutritional epidemiology.

## Declarations

### Availability of data and materials

The datasets during and/or analysed during the current study available from the corresponding author on reasonable request.
